# Children’s limited tooling ability in a novel concurrent tool use task supports the innovation gap

**DOI:** 10.1038/s41598-024-71686-8

**Published:** 2024-09-13

**Authors:** Jennifer A. D. Colbourne, Alice M. I. Auersperg, Sarah R. Beck

**Affiliations:** 1 Comparative Cognition Unit, Messerli Institute, University of Veterinary Medicine Vienna, Veterinärplatz 1, 1210 Vienna, Austria; 2 School of Psychology, University of Birmingham, Edgbaston, Birmingham, B15 2TT UK

**Keywords:** Innovation, Associative tool use, Tooling, Object relations, Spatial relations, Spatial reasoning, Psychology, Human behaviour

## Abstract

School-aged children have consistently shown a surprising developmental lag when attempting to innovate solutions to tool use tasks, despite being capable of learning to solve these problems from a demonstrator. We suggest that this “innovation gap” arises from tool tasks with more complex spatial relations. Following Fragaszy and Mangalam’s new tooling theory, we predicted that innovating a new “sticker slide” task should be more challenging when two tools need to be used at the same time (concurrently) rather than one at a time (sequentially), despite the similarity of the other task elements. In line with previous work, both versions of the task were challenging for all ages of children (4–9 years) that we tested. However, the youngest group showed particularly extreme difficulties, which was marked by not a single child innovating the concurrent version. Although success significantly increased with age, even the oldest group failed to reach 50% success on the concurrent version of the task, whereas the majority of the two older groups could solve the sequential version. Thus, in this first study of concurrent tool use in children, we found support for the prediction that increasing the complexity of spatial relations in tooling exacerbates the innovation gap.

## Introduction

Children may have all of the necessary motor skills to tie their shoelaces or braid their hair, but they are unlikely to learn to do so without repeated close observation or explicit instruction. There is a divide between the ability to engage in certain behaviours, and the capability of conceiving of them a priori. Often, it is not until certain actions have been observed or learnt from others that the possibility for action is realised. Accordingly, this is why young children benefit from instruction and guidance; complex behaviours are in a sense “jump-started” through social learning, though they may have eventually been independently discovered. Such independent problem solving, in the absence of social learning or a model solution, is known as innovation^1^.

The gap between innovating a solution unaided and being capable of learning it can vary substantially, and during development has been found to be strikingly large in the area of object relations. Bates and colleagues discovered that 10-month-old infants could only successfully solve means-end tasks if the reaching object was visibly in contact with the reward, even when the motor actions to succeed were the same in no-contact and contact tasks (e.g., placing a toy in the middle of a hoop to be pulled versus placing the toy in contact with the inside of the hoop)^2^. They concluded that the link between the two objects needed to be visually apparent in order for children to reach the conclusion that one could affect the other; the infants were incapable of perceiving a spatial relationship between the objects that did not yet exist. Subsequent research has showed that children can begin to successfully employ a reaching tool with a spatial gap around 18 months of age, although the majority could only do so after a demonstration or hint^3–5^. It is not until approximately 30 months that the majority of children systematically innovate the solution without adult aid^5,6^.

In a series of tool use studies led by Beck with young school-aged children, another surprising innovation gap in the area of object relations was discovered. This work was initially inspired by the discovery that Betty the New Caledonian crow (*Corvus moneduloides*) could innovate a hook tool by bending piece of wire in order to retrieve an out-of-reach reward inside of a vertical tube^7^, leading to the question of when such an ability emerged in children. In Beck and colleagues’ version of the task, children aged 3 to 11 years were presented with a tube in which lay a handled-basket containing a prize and given a pipe cleaner and a distractor item^8^. Surprisingly, it was not until 8 years of age that the majority of children were successful at innovating a hook tool, despite nearly all children being able to correctly select a pre-made hook tool to solve the problem, and easily able to make the requisite tool after a demonstration^8^. In subsequent experiments, various modifications to the hook task were made in order to discover which factors might influence success, but to no effect. For example, children were given experience with the bending properties of the pipe cleaner^8–10^, exploration time with the materials^10,11^, clear ownership over the tool^12^, instructions to try to make something^9^, prompts to change strategy^11^ and drawing attention from the experimenter by having a puppet present the task^9^. Even experience making another pipe cleaner tool^9^ and building a hook tool made from wooden pieces^13^ failed to increase success rates. Moreover, children’s personality traits also did not have an effect^14^. The failure to innovate on the hook task was replicated with indigenous children in South Africa, Australia and the Congo, whose developmental and cultural experiences with object manipulation vary widely from the “WEIRD” (Western, Educated, Industrialized, Rich, and Democratic^15^) populations tested by Beck and colleagues, indicating that the underlying challenge may be a cognitive one, rather than environmental^10,16,17^ (but see^12^, wherein older children visiting a museum showed higher success rates).

One suggestion is that the issue may be one of executive function, that is the cognitive abilities related to the control of behaviour. The nature of the hook task as an “ill-structured problem,” i.e., a problem lacking a clear path from start to goal, has been particularly noted as necessitating a certain degree of domain-general executive ability^1,9,11,18,19^. However, executive function and divergent thinking tasks, involving inhibition, attentional flexibility, hierarchical structuring, and working memory, have not found higher-scoring children to be better hook innovators^14,18,20,21^ (although there is evidence some of these factors may contribute to learning after a demonstration^20,21^). The problem also does not appear to be one of causal understanding, as most children can immediately select the correct tool and/or copy the manufacture of the correct tool^8,9,11^, and often innovation success rates will increase in older children after simply being shown a pre-made hook tool^19,21,22^. Remarkably, though, when given pre-made hooks that don’t fit inside the tube, either due to being oversized or having a curl at the end, children still perform poorly on the hook task^16,23^, indicating that it may be that the difficulty lies in the reshaping of the tool itself.

**Fig. 1:**
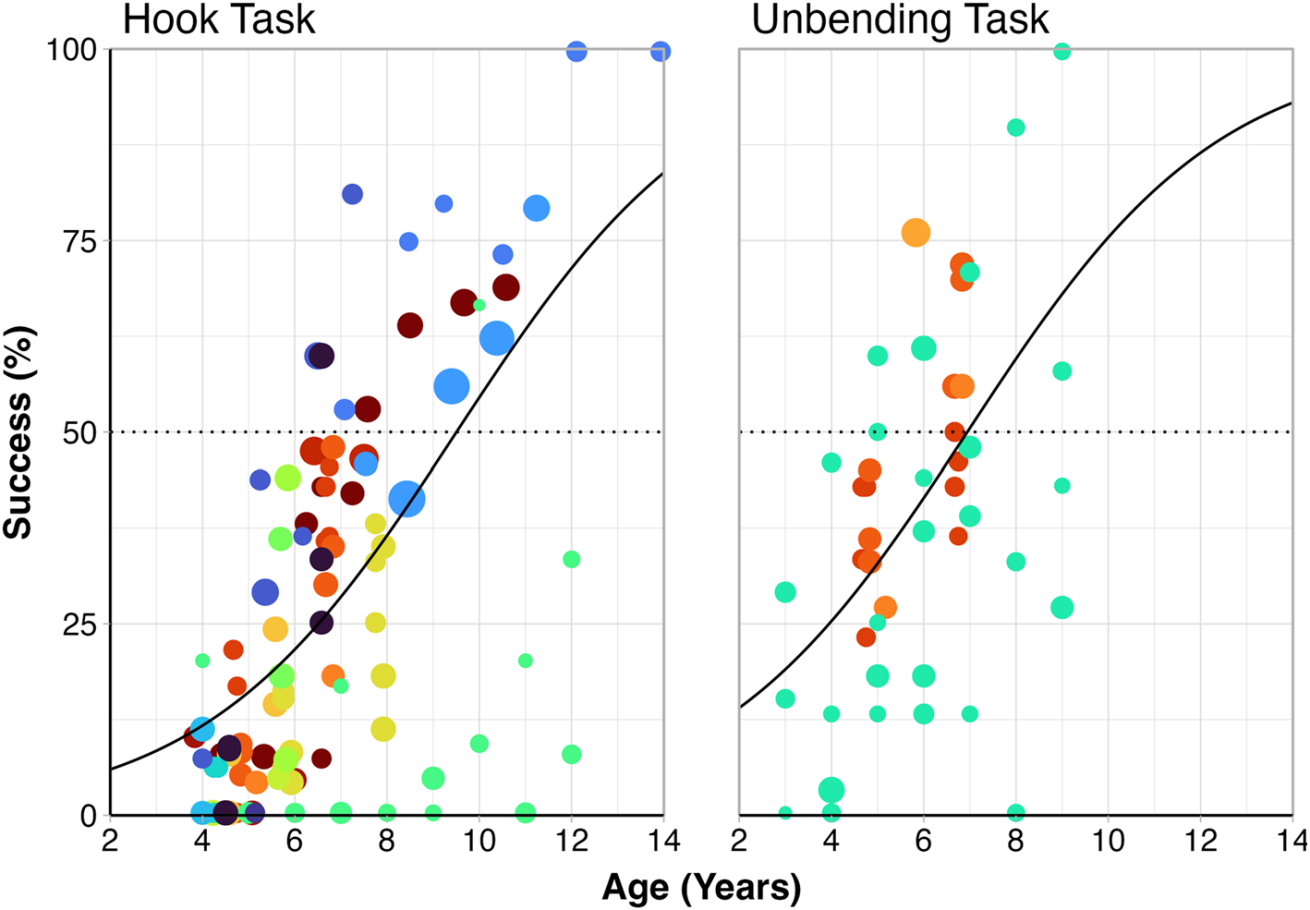
The innovation gap in two tool reshaping tasks. Each colour represents a study, each point represents an experimental condition, and sample size for each experimental condition is reflected in point size. The black line represents predicted values, weighted by sample size. The “hook task” represents only conditions in which a wire had to be reshaped; results from conditions in which children were shown a premade hook tool template before attempting the task were not included (as any subsequent tool made was not strictly innovated). More information on the experiments and data included in the graphs can be found in the open access repository.

Another tool reshaping task given to children, in which a folded wire must be unbent to push out a reward through a horizontal tube (“Unbending Task”), has been found to be easier than the hook bending task, in that it is solved on average at a younger age (7 years to reach 50% compared to 9.5 years; see Fig. 1. for predicted percentage of success based on data from previous research)^9,11,24,25^. Even with this simpler task, a similar innovation gap pattern is still apparent with higher rates of success (e.g., > 75%) only appearing late in childhood after age 10. Furthermore, children perform at significantly higher rates on this task and the hook task when they can make functional tools by adding, detaching or subtracting pieces, rather than using wire to shape a tool^26^. Building a wooden hook out of dowel pieces has also been found to be as difficult, or nearly as difficult, as reshaping a hook^13,25,27^. Thus, it may be something fundamental about making a new tool shape that is particularly difficult for young children. When considering a new tool form, one must conceive of how its new spatial relations could function to solve the problem at hand, and apply this to its creation. The challenges presented by this type of complex spatial reasoning ability could thus very well be at heart of the tool innovation issue.

Therefore, we created a tool use innovation task that eliminated tool manufacture but retained the necessity of considering novel object spatial relations in order to innovate a solution. According to Fragaszy and Mangalam’s tooling theory, it is the spatial relations involved in the act of tooling that determines the task’s complexity^28^. This is because using a grasped tool to create a mechanical effect (“tooling”) transforms the body into a body-plus-object system, in what appears to be a neurocognitively distinct phenomenon in which the body schema is extended while using a tool^29,30^. The grasped tool fundamentally alters how the actor can act in the environment as the locus of control shifts from the hand to the end-effector (distalization) and the degrees of freedom for physical movement are redistributed. Furthermore, the actor must simultaneously monitor the relationship between their grasping hand and the tool, and the effective end of the tool (the end-effector) and its mechanical interaction with the target^28^. When two tools are used in both hands at the same time, this management doubles, making it more difficult to use two tools concurrently compared to one after the other sequentially. Even without the use of tools, concurrent bimanual reaching takes longer than unimanual reaching (“two target cost”), which has been suggested to be due to the attentional demands of monitoring two objects simultaneously during sensorimotor coordination of the limbs^31–34^. Intriguingly, in tasks that require reaching bimanually for two items each placed either near or far to the participant, older children (7–10 years) exhibit a limited version of the approach taken by adults, in that movement time is increased only in congruent conditions (near/near, far/far) but not incongruent (near/far), which has been suggested to be due to the increasing sensorimotor integration that takes place during this transitional period of development^34^. They also differ from younger children (4–6 years), in that older children exhibit more adult-like synchronization, whereas younger children’s bimanual reaches are more asynchronous with weaker coupling, with the limbs almost sequential in their movements^34^.

**Fig. 2:**
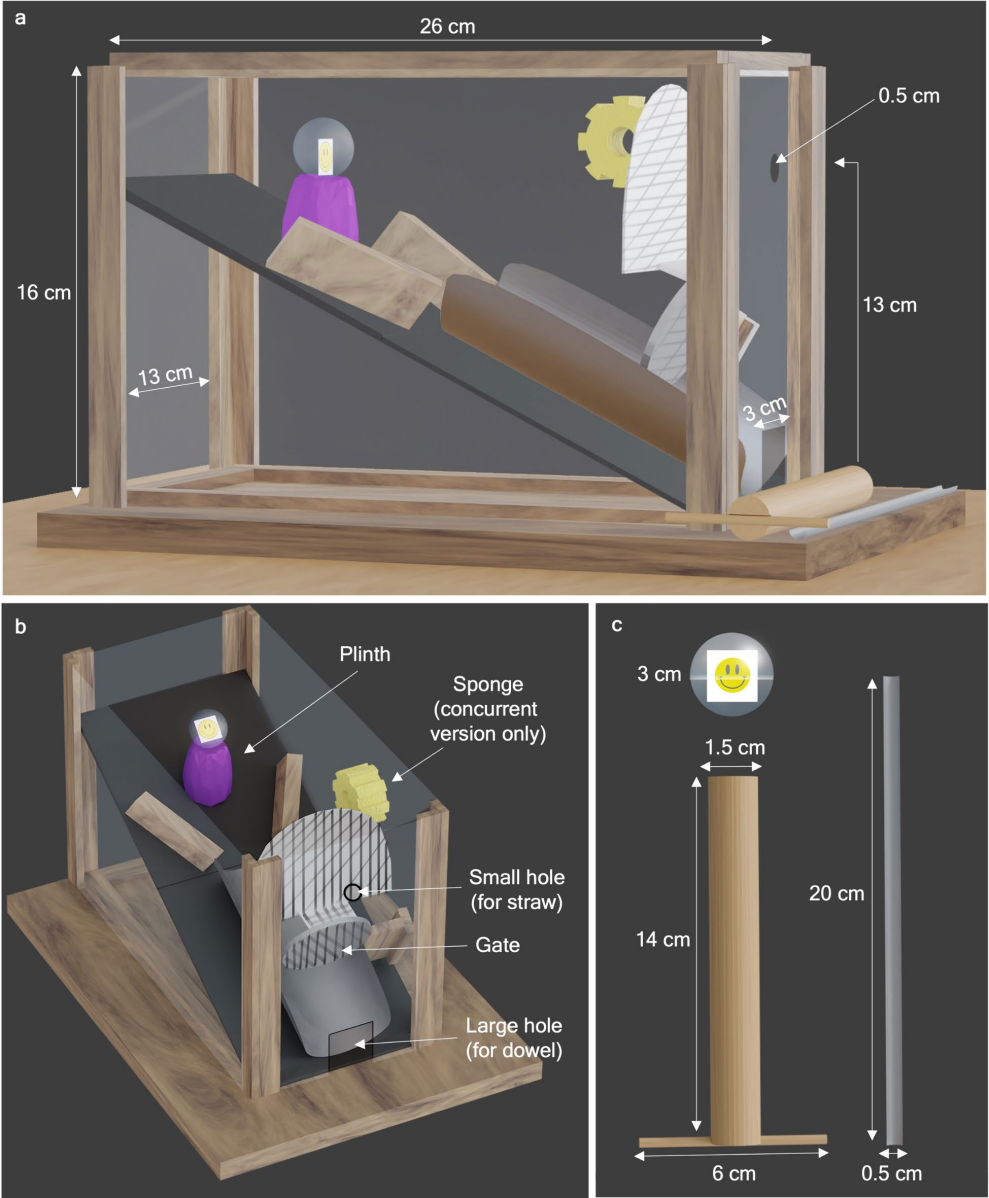
The sticker slide puzzle box. (**a**) Side view with box measurements, (**b**) high angle view with labels for essential components, (**c**) prize ball, wooden dowel tool, and thin straw tool with measurements.

Accordingly, we hypothesized that a concurrent tool use task should be more difficult to innovate than the same task with a sequential solution. We expected this difference should be more apparent in younger school-aged children, considering the innovation lag previously observed in other developmental tool innovation studies; therefore, we tested a range of children during the period of change in their tool innovation ability (4–9 years). To this end, we designed the “sticker slide” box, which has a gate at the bottom requiring a sturdy dowel to open it, that, when closed, blocks a smaller hole on top, in which can be inserted a thin straw to dislodge the reward from a platform inside (see Fig. 2). In the sequential version, the gate stays open after the dowel is inserted, allowing the apparatus to be solved in two discrete steps, whereas in the concurrent version, due to the addition of a small piece of sponge, the gate must be continually kept open with the dowel in one hand in order to probe inside with the thinner tool in the other hand (for a demonstration of each task version, see supplementary video online). According to tooling theory, the spatial relations of the concurrent version of the task should be more difficult to manage, and thus more difficult to innovate, despite each element of the apparatus remaining the same in both versions (opening the gate with one tool, dislodging the ball with the other). We expected that children who failed to innovate either solution would still be physically capable of completing the task after a demonstration and/or instruction from the experimenter, as is typical of other tool use tasks.

## Results

### Innovation success

**Fig. 3:**
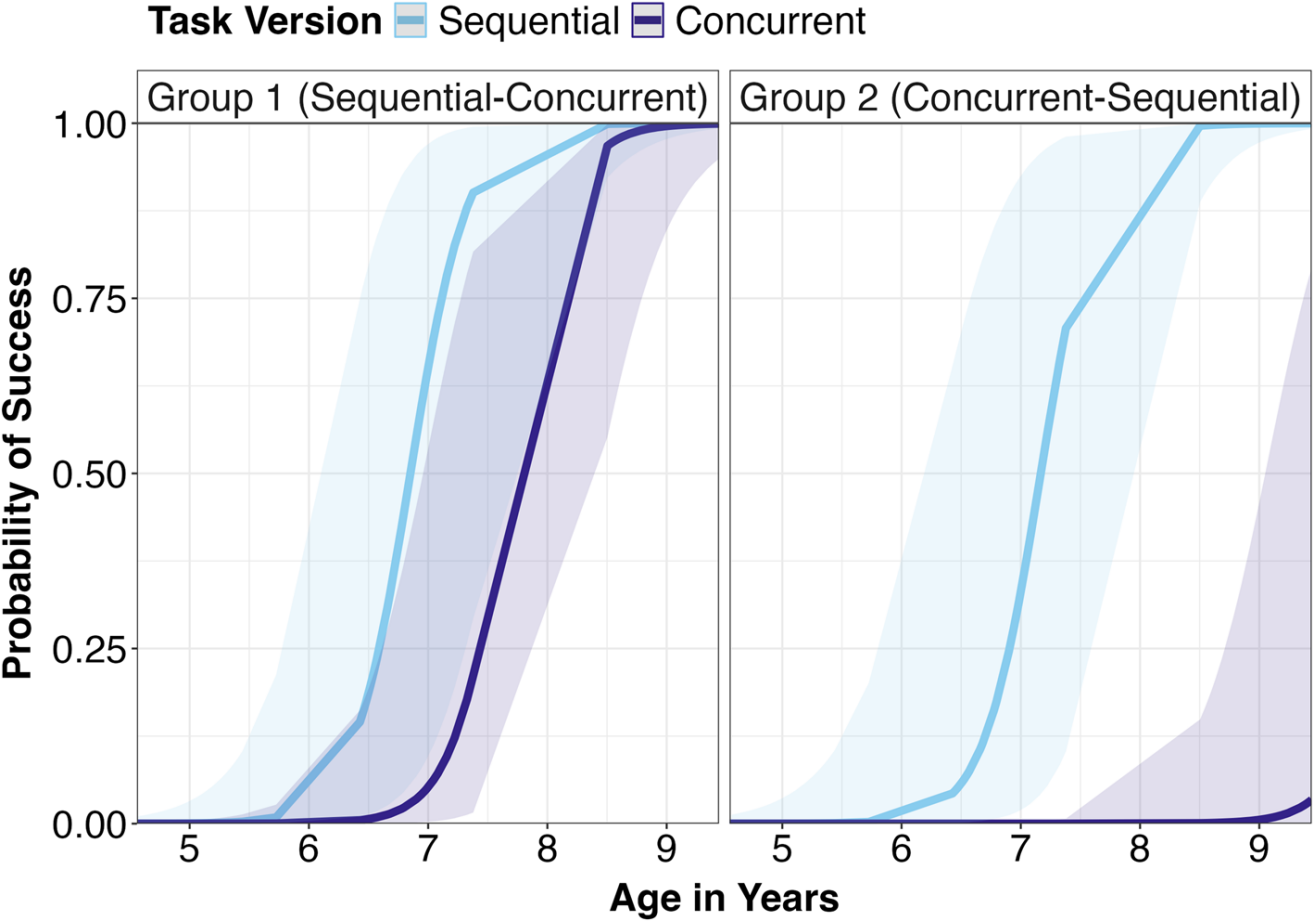
Predicted probability of success by age and task order (group). Shaded areas represent 95% confidence intervals.

We ran a logistic GLMM with success as an outcome variable (fail/success) with age and an interaction between task condition (sequential/concurrent) and task order (group 1/group 2) as fixed effects, and participant identity as a random effect. Age was z-transformed to increase interpretability of the coefficients^35^. The total number of observations was *n* = 186 (representing 98 children). We compared the full model to a null model lacking the predictor of interest (condition) using a likelihood ratio test^36^. We found that the model containing condition significantly predicted success (χ^2^(2) = 34.76, *p* < 0.001). The interaction between condition and task order was also significant (χ^2^(1) = 12.588, *p* < 0.001). These results were driven by the group that was given the sequential condition first, which was significantly more successful on the following concurrent condition (35.42%) compared to the group that was given the concurrent condition first (15.22%; *z* = 2.93, *p* = 0.018), while both groups had similar rates of success on the sequential condition (50.00% and 52.17%; *z* = 0.59, *p* = 0.937; see Fig. 3). Looking at the groups separately, the concurrent task was significantly more difficult than sequential for the group given the concurrent task first (*z* = 3.56, *p* = 0.002), but not for the group given sequential first (*z* = 2.18, *p* = 0.130). Age was significant as a main effect (see Table 1). Diagnostics of the model assumptions for collinearity, stability, distribution of the random effect and overdispersion did not reveal any cause for concern.

**Table 1:** Estimate of the success model fixed effects.

Fixed Effects	Estimate	C.I. 95%	S.E	*z*	*p*
(Intercept)	0.42	[– 11.35, 9.95]	1.45	0.29	0.771
Concurrent	– 3.52	[– 15.91, – 0.44]	1.62	– 2.18	0.030
Group 2	– 1.33	[– 18.26, 15.74]	2.26	– 0.59	0.558
Age	6.90	[1.44, 30.99]	1.62	4.25	< 0.001
Concurrent*Group 2	– 9.34	[– 42, – 0.35]	3.29	– 2.83	0.005

**Fig. 4:**
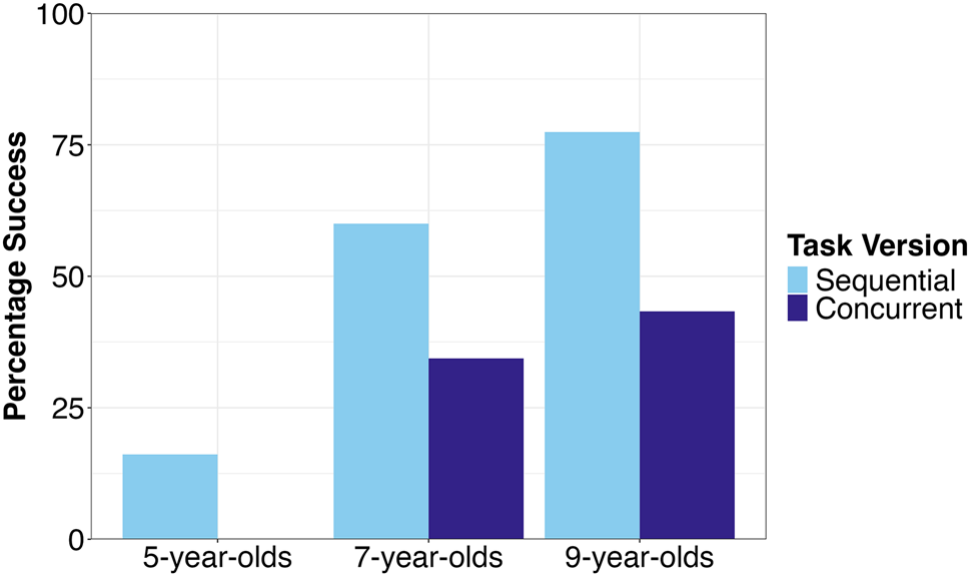
Success on each version of the sticker slide by age group.

The comparative difficulty of the concurrent version compared to the sequential version of the task can also be seen by the fact that none of the 5-year-old children (*n* = 33) managed to solve the former, compared to 16.13% of these same children successfully solving the sequential condition (see Fig. 4). Similarly, the 7-year-old (*n* = 32) and 9-year-old (*n* = 33) groups performed better on the sequential condition compared to the concurrent condition (7-year-old group: 60% vs 34.38%; 9-year-old group: 77.42% vs 43.33%); the age effect is also apparent from these numbers. Unlike the two older groups, the majority of the 5-year-olds required help during the pre-experience to familiarize them with knocking the ball off of the plinth (5-year-old group: 64%, 7-year-old group: 19%, 9-year-old group: 15%). More 5-year-olds also gave up trying to solve the puzzle in both the sequential and the concurrent versions (sequential: 16/25 children that gave up, concurrent: 15/27 children that gave up). For those children that did not succeed on the experiment and were given a demonstration afterwards, all of the 9-year-olds (18/18) were able to solve the puzzle by themselves, while 25% (8/32) of the 5-year-olds and 38% (8/21) of 7-year-olds needed a hint in order to solve the puzzle, and 41% (13/32) of 5-year-olds and 5% of 7-year-olds (1/21) needed actual physical assistance with one of the tools in order to succeed. Three children (one in the 5-year-old group, two in the 7-year-old group) never succeeded.

Of the children who were unsuccessful, 64% (29/45) tried only one tool on the sequential condition and 39% (27/70) on the concurrent condition; 71% (32/45) tried only the top hole and 2% (1/45) tried only the bottom hole in the sequential condition, and 49% (*3*4/70) tried only the top hole and 13% (9/70) tried only the bottom hole in the concurrent condition (see Table 2 for percentages by age group). In the sequential condition, 7% (3/45) of unsuccessful children succeeded in pushing the gate open but failed to solve the puzzle, and in the concurrent condition, 27% (19/70) pushed the gate open but failed. For the children given sequential first (group 1) that passed the sequential condition but subsequently failed the concurrent condition, 62.5% (5/8) discovered the gate in the concurrent condition and 37.5% (3/8) did not. We found 2% of children innovated the correct solution for the concurrent condition but failed to retrieve the ball within the time limit (one in the 5-year-old group and one in the 7-year-old group). An additional 6% were close to innovating the correct solution and engaged in concurrent tool use but did so with both tools in the bottom hole, where the straw was unable to reach the ball (three from the 7-year-old group and three from the 9-year-old group).

**Table 2:** Percentage of actions performed by unsuccessful children (with number of children in parentheses).

	Sequential Condition			Concurrent Condition		
	5-Year-Olds (*n* = 26)	7-Year-Olds (*n* = 12)	9-Year-Olds (*n* = 7)	5-Year-Olds (*n* = 32)	7-Year-Olds (*n* = 21)	9-Year-Olds (*n* = 17)
Tried Only One Tool	69% (18)	50% (6)	71% (5)	47% (15)	29% (6)	35% (6)
Tried Only Top Hole	73% (19)	75% (9)	57% (4)	56% (18)	38% (8)	47% (8)
Tried Only Bottom Hole	0%	8% (1)	0%	6% (2)	19% (4)	18% (3)
Opened Gate	0%	17% (2)	14% (1)	16% (5)	38% (8)	35% (6)

### Solving time

In order to investigate whether the concurrent condition solution took longer to innovate than the sequential condition solution, we ran a model to see if condition predicted time to success. As only children who succeeded were included in this analysis, and no 5-year-old children solved the concurrent condition, we did not include this younger age group in the model so that the five children that solved sequential did not bias the data (note that on average, the 5-year-old group in sequential performed nearly twice as slow as the older children in both conditions, *M* = 109.6 s vs *M* = 61.2 s). Consequently, the total number of observations was *n* = 66 (representing 45 children). We ran a LMM with time to success as an outcome variable with age and an interaction between condition (sequential/concurrent) and task order (group 1/group2) as fixed effects, and participant identity as a random effect. Age was z-transformed to increase the interpretability of the coefficients, and time to success was log-transformed to adjust for positive skew. We compared the full model to a null model lacking the predictor of interest (condition) using a likelihood ratio test. We found that the model containing condition significantly predicted time to success (χ^2^(2) = 14.12, *p* < 0.001). The interaction between condition and task order was also significant (χ^2^(1) = 14.12, *p* < 0.001), but not age (*t*(39.21) = 0.34, *p* = 0.736). The semi-partial R^2^ of the two interacting variables accounted for 16.9% of the variance of the response. The LMM model assumptions of normality and homoscedasticity of residuals, normal distribution of the random effect, non-multicollinearity and stability were met.

The interaction in the solving time model was driven by the fact that group 1, which received sequential first, took longer to solve the sequential condition compared to concurrent (*M* = 87.89 s vs *M* = 65.29 s), while group 2, which received the concurrent condition first, took longer to solve concurrent (*M* = 62.86 s) compared to sequential (*M* = 36.04 s) (see Supplementary Fig. S1 online). In other words, whichever condition was given first took longer to solve than the second for both groups. Note that the results are somewhat biased in that more children solved sequential (*n* = 42) than concurrent (*n* = 24); while roughly equal numbers passed sequential in group 1 (*n* = 19) and group 2 (*n* = 23), more children in group 1 passed concurrent (*n* = 17) compared to group 2 (*n* = 7).

### Dislodging time

During data collection, it was observed that there could be substantial time spent between finding the solution to the puzzle and actually dislodging the prize ball. Measuring the time from first contact of the thin straw tool with the ball and the ball being knocked off the plinth, we conducted an exploratory post hoc model to see if condition affected this dislodging time, to see if it might be the case that the motor demands of using two tools concurrently with two hands are more difficult than when using only one hand to manipulate one tool. As with the success time model, only children who actually solved the puzzle were included in this model, so the data was limited only to the children in the 7-year-old group and the 9-year-old group (the five 5-year-old children in sequential were removed again to avoid biasing the data). Furthermore, a two-part hurdle-style model had to be employed as roughly half of the children immediately knocked the ball off of the plinth, resulting in an uneven distribution due to excess zeroes. The time to dislodge data was log transformed due to positive skew. Full-null comparisons revealed neither the zero part (χ^2^(1) = 0.440, *p* = 0.507) nor the remainder of the model part (χ^2^(1) = 0.012, *p* = 0.913) were significant. This is reflected in the fact that roughly equal numbers of children immediately solved the puzzle as those that required more time, in both the sequential (20 vs 22) and concurrent conditions (10 vs 14). Similarly, the amount of time taken to dislodge the ball was similar in both conditions (sequential: 20.68 s; concurrent: 21.57 s). Diagnostics for the assumptions of both models were acceptable, however the random effect of participant identity was overfitted.

## Discussion

Similar to prior work, we found a lag in children’s ability to innovate. Even though the sequential version of the sticker slide task could technically be solved without a deeper understanding of the function of the gate and tools (i.e., by inserting the various tools in the holes until the gate opened/ the prize ball was dislodged), not even a quarter of the youngest group was successful (compared to over three-quarters of the oldest group). Remarkably, not a single child of the younger group succeeded in innovating the concurrent version. Even after a demonstration by the experimenter, most of the 5-year-old group required help in the form of a hint or even physical assistance to succeed. This was needed by none of the oldest age group. This is in contrast to the hook manufacture tasks, where some children as young as three years can make a hook and solve the puzzle after a single demonstration. The concurrent condition was more difficult to innovate than the sequential condition for the older children, as the oldest group of 9-year-olds only reached a success rate of 43.33%, which is remarkably lower than the success rate of the tool reshaping tasks for that age. Their success rate for sequential, however, is much more comparable (77.42%) to the hook and horizontal tube tasks.

For the older two age groups, the amount of time required to innovate and successfully solve the puzzle in both conditions only depended on which condition had been given first. And so, whereas the concurrent condition may be harder to innovate, for those children that do innovate, there does not appear to be a difference in solving time between the two conditions. Similarly, there did not appear to be a difference between conditions when looking at the time to knock the ball off the plinth, after contact was made with a tool. These results hint that the chief difficulty is making the innovation itself, rather than a more physical element, such as motor coordination. Of the children who tried only one hole, the large majority focused only on the top hole rather than the bottom. It is possible that this is because the top hole was directly in the children’s line of sight when seated, and/or that the children were influenced by the location of the hole in the pre-experience box and persisted in this approach due to a kind of functional fixedness. Considering two-thirds of the children who were unsuccessful on the sequential condition only tried one tool, this further suggests persistence with a single approach as a cause of failure.

However, for both models, the data was limited to only the successful children, of which there were many more in the sequential than concurrent condition, and also resulted in no representation from the youngest group. Considering the difficulty this group had completing the concurrent version after the demonstration, potential differences in motor demands between simultaneous and concurrent tool use for younger children cannot be dismissed. It is also possible that the difference is relatively small, requiring a more sensitive methodology to measure. For example, in one key pressing study, the difference between unimanual and bimanual responses in children differed only by milliseconds^37^. Interestingly, the researchers found bimanual responses took significantly longer than when responding to the same stimulus unimanually, but only once the children reached six years of age^37^. This phenomenon, known as “bimanual cost,” also occurs in adults, and has been suggested to be caused by inhibitory networks in the corpus callosum that slow the response of each hemisphere when coupling bimanual responses^37,38^. Consequently, its absence in younger children suggests that this rudimentary form of interhemispheric communication supporting bimanual movement has not yet developed^37^. Similarly, when reaching to grasp with both arms simultaneously, compared to older children (7–10 years), younger children (4–6 years) show immature patterns of bimanual coordination, with each limb moving in a more sequential manner, resulting in weaker spatial and temporal coupling^34,39^. Bimanual tasks that require coordinating two distinct actions (role differentiated bimanual movements) also show marked differences in movement time and smoothness between 5–6 years and 7–9 years^40^.

The gradual maturation of the corpus callosum has been suggested to be an important factor in the development of both interhemispheric integration and bimanual coordination^40,41^. The callosal isthmus, which connects temporo-parietal areas associated the understanding of spatial relations and language, exhibits rapid growth between 7 and 11 years^42^, as well as functional-structural reorganization and refinement during age 6 to 8^43^. Immature bimanual coordination may explain why some of the younger children in our study needed physical assistance with one of the tools in order to eventually dislodge the prize. However, considering there were only two cases of children correctly innovating the solution but failing to dislodge the ball in time in the concurrent condition, it does not seem likely that the issue was one of bimanual coordination per se. However, lack of sensorimotor integration between the two hemispheres certainly could impede the children’s ability to consider prospective concurrent motor solutions. It is certainly intriguing, and likely a fruitful area for subsequent research, that eight years of age has been argued to represent a developmental milestone in motor efficacy and sensorimotor integration^34,44,45^, which is around the same time children have been found to improve at tool innovation tasks (as shown in Fig. 1).

This is the first study to look explicitly at the innovation of concurrent tool use in children. Previously, there have been a few studies of sequential tool use in children under six years of age, all of which involve using a tool to obtain a different tool that can access a reward, with success rates ranging from 5.1 to 37.5%, depending on the amount of pre-experience the children had with the tool elements^46–48^. In line with these low success rates, the youngest group in our study (5-year-olds) was only 16% successful. Even when requiring only one tool, solving a puzzle with two different actions is also difficult for older children; in a task requiring a rod to push an object inside a box then rake it out (“chimney task”), just over half of the 7-year-olds tested were correct, whereas on a task requiring the use of a rod first as a lever to bring the object closer, then next extracting the object (“sloping task”), it was not until 10½ years of age that just over half were successful^49^. Clearly, using multiple tools or the same tool for multiple functions is a developmental challenge.

This type of tool use, referred to as associative tool use in the non-human animal literature^50,51^, is not only difficult for young humans. For example, the use of the same tool for different functions (multi-functional tool) has only been repeatedly observed in chimpanzees (*Pan troglodytes*)^52^. Although there are more cases of sequential tool use, it is limited to only a few highly intelligent animal species (e.g., great apes^53,54^); tufted capuchin monkeys (*Sapajus spp.*)^55^; Goffin’s cockatoos (*Cacatua goffiniana*)^56^; New Caledonian crows (*Corvus moneduloides*)^57^; rooks (*Corvus frugilegus*)^58^. To date, there are no studies with animals engaged in concurrent tool use, and Fragaszy and Mangalam have hypothesized that simultaneously tooling with two grasped objects is beyond the capacity of any animal species^28^.

In comparison to associative tool use, success rates of single-step tool use tasks are typically much higher and within the capability of children under three years of age (e.g. spoon feeding, hammering pegs, raking in an out of reach object, as well as various probing tasks in the great ape tool test battery (GATTeB)^5,59–61^. Notably, the one task that children found especially difficult to innovate in the GATTeB was “nuthammer,” in which a child needed to use a clay hammer to crack open a plastic nut; the experimenters thought this task was particularly demanding because it involved multiple steps and the coordination of several objects (hammer, anvil and nut)^61,62^. This type of associative tool use, also referred to as composite tool use^50,51^, is similarly challenging for animals. The use of a hammer and anvil is limited to a few primate species (e.g. chimpanzees (*Pan troglodytes*)^51^; long-tailed macaques (*Macaca fascicularis*)^63^; capuchin monkeys^64^). In this case, two spatial relations need to be managed in a sequence (nut-to-anvil, hammer-to-nut). The only other comparable tool use task to date is a laboratory study with Goffin’s cockatoos, in which a ball needs to be placed inside a box, then maneuvered with a stick in order to collapse a platform (“golf club task”)^65^. Success on this task depends on an external-concurrent interaction, in which two objects are simultaneously used as the positioning and movement of the ball is controlled by the stick^65^. This task is the closest in spatial relations management to the concurrent tool use task of the sticker slide, and in parallel to our own results, only three of the cockatoos were capable of consistently solving the task, out of the eleven capable of using tools to solve simpler tasks.

It can thus be seen, in both children and animals, that the increased number of spatial relations as well as the temporal order of managing these relations sequentially or concurrently affects the success rate of tool use innovation tasks. As predicted by tooling theory, we found that innovating concurrent tool use was considerably more difficult than sequential tool use. In the sequential tool task, it is possible for the solution to be fortuitously discovered while exploring by inserting dowel in the bottom hole, without necessitating a deeper understanding of the function of the gate or the spatial relations involved. In contrast, the concurrent tool task requires considering the prospective spatial relations concerning the gate and the provided objects, in addition to keeping the gate open to allow access to the plinth while simultaneously monitoring both tools. The higher success rates of the group that received sequential first, however, indicates that discovery of the gate’s affordances may have facilitated performance in the concurrent condition. Still, it is telling that although some of the youngest children were successful on the sequential version, none of them were able to solve the concurrent condition, despite those in the group given sequential first ostensibly realising the gate could open. Moreover, nearly a third of children in the concurrent condition pushed the gate open and still failed, indicating that at least some had an idea of how the gate functioned. It is also difficult to ascertain how much information about the gate was learned by probing with the straw, as the gate would move to at least some degree when pushed, which should have indicated that the gate was not immoveable. Several authors have pointed to the failure to integrate or detect potential relationships between objects and their affordances may be what makes tool use challenging^66,67^. Similarly, the hook bending task requires perceiving spatial relations that do not yet exist, in this case of an entirely new tool. It would be beneficial for future work to explore the potential link between affordance learning and prospective perception, especially with different tasks that have no components that can be solved without a deeper understanding of the relations between objects. It would also be informative to look into the relationship of bimanual coordination and the development of the corpus callosum with the ability to succeed in and innovate concurrent tool use tasks.

Our research confirms that the innovation gap in children exists beyond tool reshaping tasks. We suggest that prospective perception of spatial relations may be the limiting factor for children’s innovative abilities, explaining why children can still readily learn the task after social demonstration. As with very young children that can only succeed in pulling a spatially distant object with a rod once they have been shown the possible physical relationship by an adult, older children also struggle with multiple spatial relations in complex tooling tasks until they have been revealed. This is not necessarily a drawback, as learning tool use socially at a young age may be more efficient and productive than completely “reinventing the wheel”^1,8^. Other research has shown that similarly aged children struggle with considering the near future, such as the representation of mutually exclusive possibilities^68–71^, and it has been suggested that foresight may play a role in innovation^72^. It is premature to do more than speculate whether these phenomena are related without more empirical support, but it is telling that in both cases young children struggle with conceiving of yet non-existent future possibilities. The innovation gap may not simply be a failure of complex problem solving, but a failure of imagination.

## Methods

### Participants

Children were recruited from two primary schools located in two ethnically diverse areas in Birmingham, England, serving low- and middle- income families. The final sample consisted of 98 children (49 males, 48 females, 1 unspecified) between the ages of four and nine (range: 55 mo.–113 mo.) from three school years (5-year-old group: *n* = 33, *M* = 60 mo.; 7-year-old group: *n* = 32, *M* = 81 mo.; 9-year-old group: *n* = 33, *M* = 108 mo.). This excludes the data for 7 children that solved the puzzle box in an unintended manner on both conditions (e.g., throwing objects, shaking the box etc.).

### Ethics

Ethical approval was granted by the University of Birmingham, UK, STEM Ethical Review Committee, and research was conducted in accordance with all relevant guidelines and regulations. Informed consent was obtained from each participant’s parent or legal guardian prior to participation in the study.

### Materials

The “sticker slide” is a Plexiglas box with a half of a pipe on a ramp and a plinth where a prize ball (a clear plastic ball that could be opened to obtain a sticker) can be placed. There is a wide gap in the Plexiglas at the bottom of the ramp for the ball to exit the pipe, and a small hole in line with the plinth, wide enough only to fit a slim tool. Blocking the path of the pipe is a hinged gate, which can be pushed open with a sturdy tool. In the concurrent version of the task, a piece of sponge was placed on the wall to prevent the gate from staying open without consistent pressure from the tool.

The tools provided for the experiment consisted of a thin flimsy tool (a third of a plastic straw), and a short sturdy tool (a thick wooden dowel with a cross piece attached to one end), in order to prevent the dowel from being pushed completely into the bottom hole of the apparatus. This crosspiece was added to the sturdy tool after the first session, as three children attempted to “throw” the dowel inside the box.

The pre-experience puzzle box was a simplified version of the sticker slide, consisting of only a ramp with a gap at the bottom, and a small hole in line with the plinth that could fit the 0.5 cm diameter wooden stick provided. The pre-experience box was a warm-up with no time limit employed to ensure that the children knew the affordance of a stick tool for reaching, that they had permission to use tools to solve the tasks, and to make them comfortable with the experimenter and the experimental situation.

### Procedure

Before testing, the teacher instructed the children not to tell the other children how to solve the puzzles in order to “keep the activity a fun surprise.” Only children given prior parental permission were allowed to participate. All participants were tested by a female experimenter (J.A.D.C.) in an open quiet room near the classroom and their performance video-recorded. Children were alternately assigned to group 1, which received the sequential version of the task first and the concurrent version second, and group 2, which received the concurrent version first and sequential version second.

Once the child entered the testing room, they were given a bookmark and then asked if they would like to solve some puzzles in order to obtain stickers to put on the back. The children were asked to first sit at a table holding the pre-experience puzzle box. The experimenter pointed out that there was a sticker contained in the clear prize ball sitting on the platform. The child was asked if they thought they could retrieve the ball, and informed that they could use anything on the table to do so. They were further instructed not to take off the lid or try to hit the box. Children that initially failed to solve the task were given a “hint,” either that they should try the stick or that there was a hole at the top, depending on their difficulty with the task. The 5-year-old group more often needed a hint (64%, 21/33), compared to the two older groups (7-year-olds, 19%, 6/32; 9-year-olds, 15%, 5/33).

The child was then asked if they would like to try another puzzle. They were asked to sit at a different table with the testing box. The experimenter repeated the instructions from the pre-experience. If, during a trial, the child appeared to give up or stated that they could not do it, then they were asked if they would like to keep trying. If they answered no, the trial ended. Otherwise, a trial ended after the child solved it or three minutes elapsed. The next trial began by the experimenter informing the child that they were going to make one little change, and asked if the child wanted to try again. The experimenter then either removed the sponge that kept the gate from staying open (for the sequential version) or added the sponge (for the concurrent version), and the child was told they could start again.

At the end of the testing session, children that could not solve either the sequential or concurrent trials were told that the experimenter would show them how to solve it and then given a full demonstration, and asked if they wanted to give it another try. If they still struggled, the experimenter either gave them a hint if they had figured out the function of both tools and were close to solving it (usually to apply more pressure to keep the gate open) or helped them to solve the puzzle by assisting with either the dowel or straw (whichever was causing the child more problems). In this way, all children who participated received all of the sticker rewards.

### Analysis

We recorded several outcome variables, including whether the child succeeded or failed in each condition, how long it took them to solve the puzzle (from coming in contact with the tools until the ball was dislodged from the plinth), how long it took the child to dislodge the ball from the plinth (once first contact was made with the prize ball), whether the child needed help in the pre-experience, or whether after the demonstration the child required a hint or physical help to solve the puzzle. After a suggestion by a reviewer, we had the videos re-coded to determine how many tools the child used to try and solve the puzzle, which holes they interacted with (top/bottom), whether they pushed the gate open, and which children tried concurrent tool use but did not succeed within the time limit.

All analyses were conducted in R version 4.1.1^73^ with an alpha level of 0.05. Generalized linear mixed models (GLMMs) were run using the lmer/glmer functions of the lme4 package version 1.1–27.1^74^. Semi-partial R^2^ were calculated using the r2glmm package version 0.1.2^75^. Variance inflation factors (VIFs) were calculated using the car package version 3.1–2^76^. The remaining model diagnostics and confidence intervals were estimated with functions kindly provided by Roger Mundry.

## Supplementary Information

Supplementary Video 1.

Supplementary Information 1.

## Data Availability

All data and analyses are freely available at the Open Science Framework (OSF): https://osf.io/hrmx4/.
