# Supplementary material for: Children’s limited tooling ability in a novel concurrent tool use task supports the innovation gap
Source: Sci Rep. 2024 Sep 13;14:21374. doi: 10.1038/s41598-024-71686-8 (PMC11393408; doi:10.1038/s41598-024-71686-8)
Supplement: Supplementary file 2 — Supplementary Information 1. [file 41598_2024_71686_MOESM2_ESM.pdf]

Supplementary Information

Evidence for the innovation gap:
Limitations in children's tooling abilities found in a novel concurrent tool use task

Jennifer A. D. Colbourne, Alice M. I. Auersperg, Sarah R. Beck

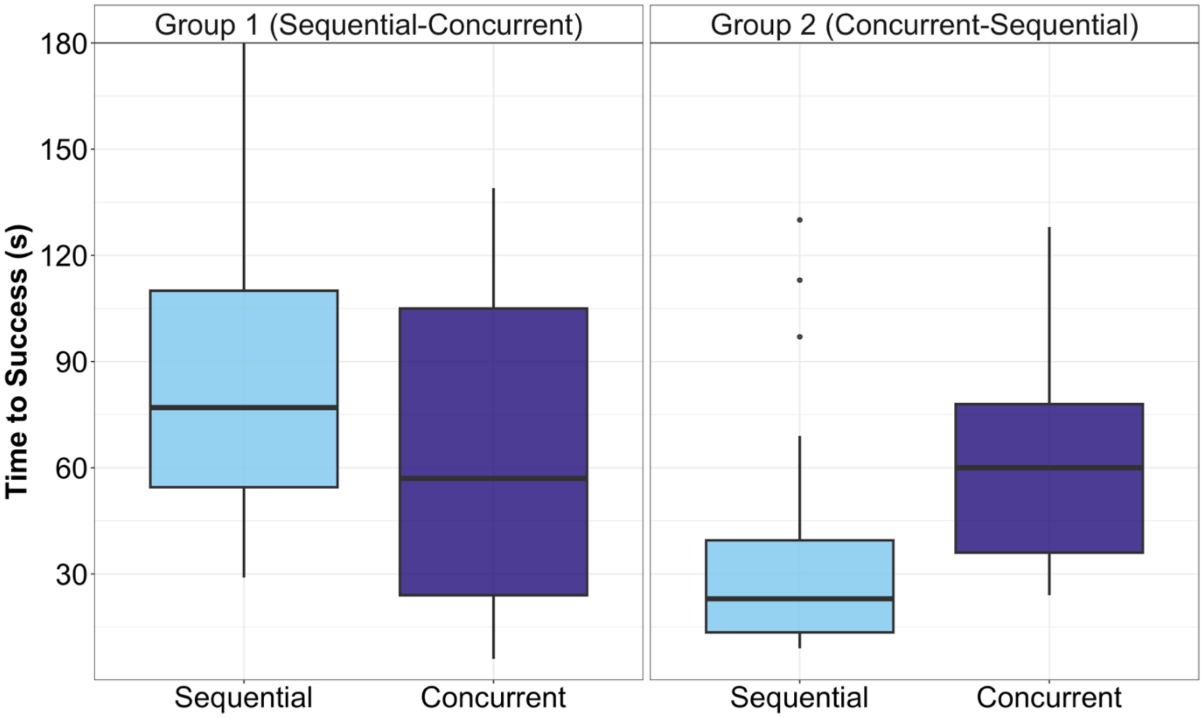

Supplementary Figure S1. Time to success by task order (group). The data from the youngest group (only of five of which succeeded on sequential) is not included.

Supplementary Video S1. Example of the tooling actions required to solve the sticker slide apparatus in the 1) sequential condition (6-18 s), and 2) the concurrent condition (19-34 s).
